# Urticaria

**DOI:** 10.1186/s13223-024-00931-6

**Published:** 2024-12-09

**Authors:** Moshe Ben-Shoshan, Amin Kanani, Chrystyna Kalicinsky, Wade Watson

**Affiliations:** 1 Division of Pediatric Allergy, Clinical Immunology and Dermatology, Department of Pediatrics, McGill University Health Center, Montreal, QC Canada; 2 Division of Allergy and Immunology, Department of Medicine, University of British Columbia, Vancouver, BC Canada; 3 Section of Allergy & Clinical Immunology, Department of Internal Medicine, University of Manitoba, Winnipeg, MB Canada; 4 Division of Allergy, Department of Pediatrics, IWK Health Centre, Dalhousie University, Halifax, NS Canada

**Keywords:** Urticaria, Acute urticaria, Chronic urticaria, Chronic spontaneous urticaria, Inducible urticaria

## Abstract

Urticaria (hives) is a common disorder that may be associated with angioedema (swelling that occurs beneath the skin). It is generally classified as acute or chronic, and chronic urticaria is further classified as spontaneous or inducible Second-generation, non-sedating histamine type 1 (H1)-receptor antihistamines represent the mainstay of therapy for both acute and chronic urticaria. Second-line treatment for uncontrolled chronic urticaria includes omalizumab (a monoclonal anti-immunoglobulin E [IgE] antibody). In this article, we review the causes, diagnosis and management of urticaria (with or without angioedema).

## Key take-home messages

- Urticaria is a common disorder characterized by recurrent, pruritic (itchy) lesions with pale centers (wheals) that usually subside within 48 h; it can be associated with angioedema.
- Mast cells are the primary effector cells in urticaria.
- Urticaria is classified as acute (lesions for < 6 weeks) or chronic (lesions for ≥ 6 weeks). Chronic urticaria can be further classified as spontaneous or inducible.
- The diagnosis of urticaria is based primarily on a thorough clinical history and physical exam; however, diagnostic tests may be helpful in some instances.
- Second-generation, non-sedating H1-receptor antihistamines are the mainstay of therapy for urticaria. Omalizumab and cyclosporine can be used for more severe, chronic cases.
- Referral to an allergy specialist or dermatologist should be considered in the following situations: failure of or difficulty tolerating first-line second-generation antihistamine treatments; need for specialized treatment; and the presence of severe symptoms and/or atypical features.

## Introduction

**Fig. 1:**
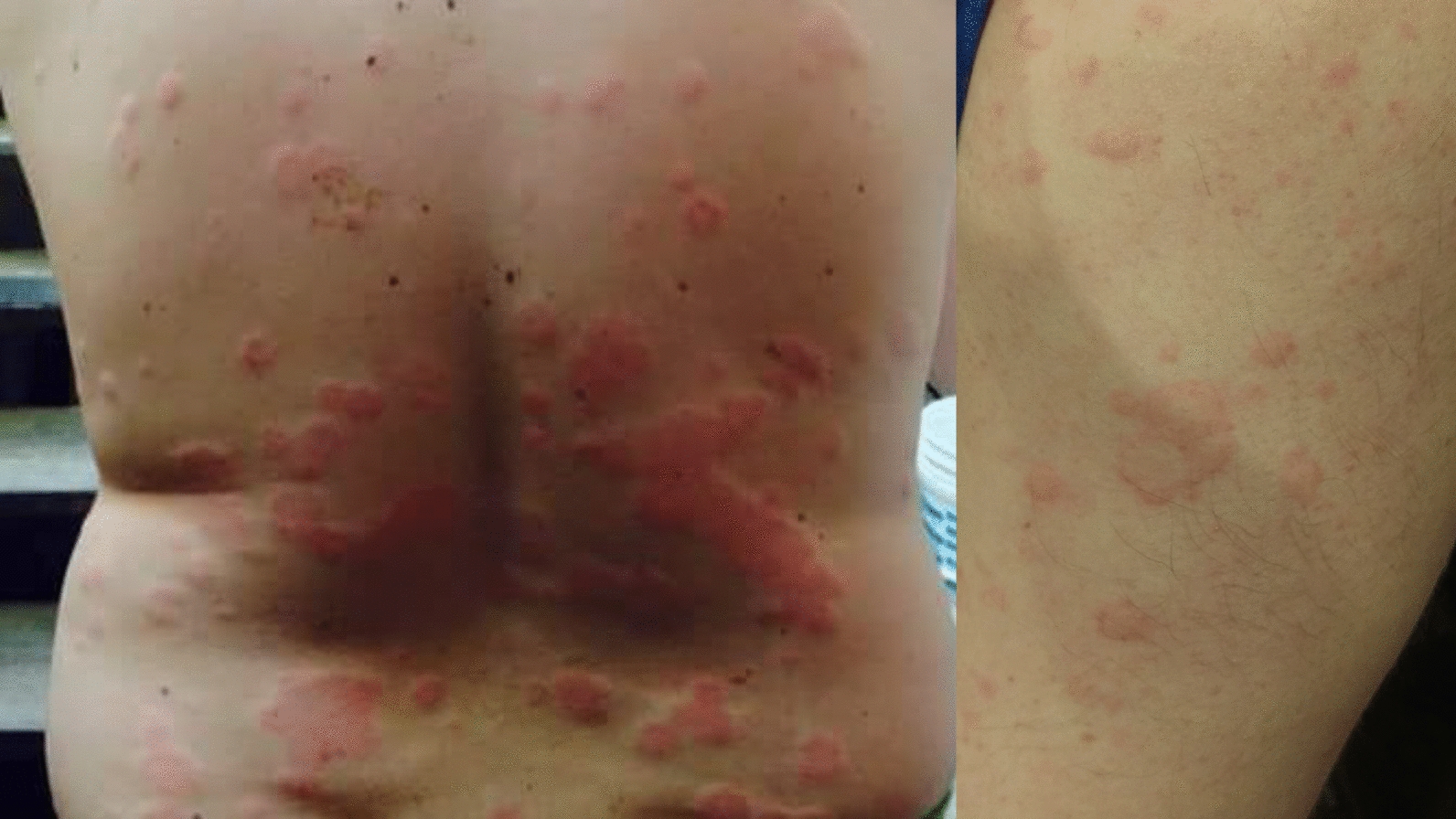
Urticaria (hives)

Urticaria is a common disorder, occurring in 15–25% of individuals at some point in life [1, 2]. It is characterized by recurrent, pruritic wheals with pale, central swelling and surrounding epidermal erythema which can appear over any part of the body (see Fig. 1). The lesions can range in size from a few millimeters to several centimeters in diameter, and are often transient, resolving within about 24 h without scarring; however, some lesions may last up to 48 h [1–4]. Angioedema (swelling that occurs beneath the skin) is reported in 40% to 60% of patients with urticaria [5].

Mast cells are the primary effector cells in urticaria and in many cases of angioedema (see *Angioedema* article in this supplement). These cells are widely distributed in the skin, mucosa, and other areas of the body, and have high-affinity immunoglobulin E (IgE) receptors. Mast cell degranulation leads to the rapid release of various inflammatory mediators, such as histamine, leukotrienes and prostaglandins which, in turn, cause vasodilation and leakage of plasma in and below the skin. There is also a more delayed (4–8 h) secretion of inflammatory cytokines (e.g., tumor necrosis factor, interleukin 4 and 5) that potentially leads to further inflammatory responses and longer-lasting lesions [1].

**Fig. 2:**
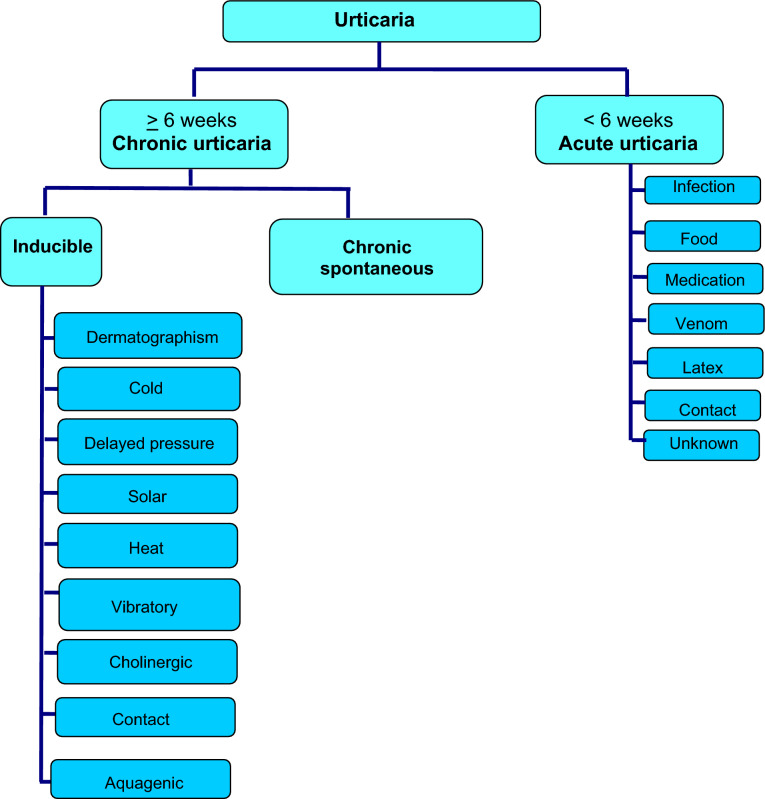
Classification of urticaria

Urticaria is generally classified as acute or chronic, depending on the duration of symptoms and the presence or absence of inducing stimuli (see Fig. 2) [6]. Acute urticaria refers to urticaria which lasts less than 6 weeks. Chronic urticaria is defined as urticaria, angioedema or both that has been continuous or intermittent for at least 6 weeks [6]. Chronic urticaria can be further classified as chronic spontaneous urticaria (CSU) or inducible urticaria. The latter represents a distinct subgroup of chronic urticaria that is induced by physical stimuli, such as scratching (dermatographism, a common form of physical urticaria), cold, heat, sunlight, vibration and pressure.

Although acute urticaria can generally be easily managed and is associated with a good prognosis, chronic urticaria is often associated with significant morbidity and diminished quality of life (QOL) [7]. Inducible urticaria also tends to be more severe and long-lasting, and can sometimes be challenging to treat [1, 3].

This article focuses on the causes, diagnosis and management of the most common types of urticaria.

## Classification and etiology

### Acute urticaria

The most common causes of acute urticaria (with or without angioedema) are medications, foods, viral infections, stress, parasitic infections, insect venom, and contact allergens (e.g., latex) [8]. Medications known to commonly cause urticaria with or without angioedema include antibiotics (particularly beta lactams and sulfonamides), non-steroidal anti-inflammatory drugs (NSAIDs), acetylsalicylic acid (ASA), opiates and narcotics. The predominant foods that cause urticaria are milk, eggs, peanuts, tree nuts, fish, and shellfish. Viral infection is another potential trigger and can be the primary etiologic agent causing acute urticaria [9]. In these cases, urticarial manifestations resolve when the infection heals or is controlled. Acute urticaria should be differentiated from anaphylaxis which has similar triggers including food, medication, and insect stings; however, treatment approaches will be different (see *Anaphylaxis* article in this supplement). Up to 36% of patients with acute urticaria can progress to chronic spontaneous urticaria (CSU) [10].

### Chronic urticaria (CU)

A recent systematic review and meta-analysis reported an overall lifetime CU prevalence of 4.4% and an overall point prevalence of 0.7%, ranging from 0.1% in North America to 0.5% in Europe, and up to 1.4% and 1.5% in Latin America and Asian countries, respectively [11]. CU has increased over the last decade, with studies suggesting a 2- to 10-fold increase in prevalence [11, 12]. The prevalence of isolated angioedema in the context of CU has not been studied, but it is considered to account for less than 10% of CSU cases [5]. CU is reported to affect primarily adults, with a peak age of onset between 20 and 40 years, and it affects women more frequently than men [3, 5, 13, 14]. However, recent studies suggest that children, adolescents and geriatric populations may also be affected to a similar extent. Prevalence rates of 1% and 1.4% have been reported in children under 14 years of age and in those under 18 years of age, respectively [5, 15, 16].

In CSU, an external trigger cannot be identified (see Fig. 2). Almost half of these cases are thought to occur due to pathogenic autoimmune mechanisms involving either immunoglobulin G (IgG)- or IgE-mediated pathways [17]. In the former, circulating IgG autoantibodies that recognize IgE antibodies or the alpha subunit of the high-affinity IgE receptor on dermal mast cells and basophils lead to chronic stimulation of these cells and the release of histamine and other inflammatory mediators that cause urticaria and angioedema. In the latter, IgE directed against a variety of auto-allergens promote mast cell/basophil degranulation [17, 18].

CSU is also associated with autoimmune thyroid diseases [19, 20], and antithyroid antibodies have been observed in approximately 27% of CSU cases [13, 14]. Numerous other autoimmune disorders, including rheumatoid arthritis, systemic lupus erythematosus (SLE), dermatomyositis, polymyositis, Sjogren’s syndrome, and Still’s disease have been associated with CSU [21]. Investigations for these conditions are not warranted unless there are clear features of their presence on clinical evaluation.

A variety of chronic infections have also been reported to be associated with CSU, including viral hepatitis B and C, Epstein-Barr virus, herpes simplex virus, *Helicobacter pylori* infections, and helminth parasitic infections. With the exception of parasitic infections, these are not believed to play a significant role in most cases of CSU. A systematic review on etiological factors associated with CSU in children showed that infections could be associated with 1% of cases, and parasites with 3.5% [22].

### Inducible urticaria

Chronic inducible urticaria (CIndU) is elicited by a specific trigger (Fig. 2) [6]. The most common type of CIndU is dermatographism (also known as “skin writing”), in which lesions are created or “written” on the skin by stroking or scratching the skin. Pressure areas from clothing such as the waistline (i.e., after wearing tight-fitting pants) and the area of the ankles or calves that makes contact with the elastic band of socks are commonly affected [1–3, 23]. A statistically significant correlation has been reported between urticaria and dermatographism in the pediatric population, without any differences between the acute and chronic forms. Consequently, it has been suggested that dermatographism could be related to urticaria per se and not only to the chronic inducible form, suggesting a unique intrinsic marker in all forms of urticaria [24].

Cold urticaria is a type of CIndU characterized by pruritic wheals and/or angioedema that is triggered by skin exposure to cold air, liquids, or objects. The hives/angioedema commonly occur upon rewarming of the skin. The pooled prevalence of cold urticaria among patients with CU and CIndU is 7.62% and 26.10%, respectively [25]. Up to 20% of patients with cold urticaria may experience anaphylaxis, mainly after swimming in cold water [25].

Cholinergic urticaria results from a rise in basal body temperature that occurs following physical exertion or exposure to heat, and it is reported to affect 0.025% of the population [12]. Other physical stimuli which can trigger urticaria include exposure to ultraviolet light (solar urticaria), water (aquagenic urticaria), and vibration. The lesions produced by these physical stimuli are typically localized to the stimulated area and often resolve within 2 h. However, some patients may experience delayed-pressure urticaria which, as the name implies, comes on slowly (i.e., 30 min to 12 h) after pressure has been applied, and can last several hours or even days. Typical areas affected include the hands and feet, especially when constant pressure is applied to these areas during specific tasks or in certain occupations.

## Diagnosis

The diagnosis of urticaria is based primarily on a thorough clinical history and physical examination. Based on the history and physical exam, diagnostic tests may also be considered to help confirm a diagnosis of acute, chronic or inducible urticaria.

### History and physical examination

The history and physical examination should include detailed information regarding: the frequency, timing, duration and pattern of recurrence of lesions; the shape, size, site and distribution of lesions; potential triggers (e.g., food, medications, physical stimuli, infections, insect stings, stressful occurrences); response to previous therapies used; and a personal or family history of atopy [6, 13, 14]. Many conditions can easily be confused with urticaria, particularly urticarial vasculitis and systemic mastocytosis (see Table 1 for conditions that need to be considered in the differential diagnosis of urticaria). In urticarial vasculitis, the lesions are usually painful rather than pruritic, last longer than 48 h, and leave bruises or discoloration on the skin [1, 26]. Systemic mastocytosis (also called systemic mast cell disease) is a rare condition that involves the internal organs, in addition to the skin. In this disorder, atypical mast cells collect in various tissues that can affect the liver, spleen, lymph nodes, bone marrow and other organs [1]. Initially, urticaria can be confused with erythema multiforme (and vice versa) but the latter develops quite differently with discrete targetoid lesions that do not occur in urticaria [27].

**Table 1:** Conditions to consider in the differential diagnosis of urticaria

Urticarial vasculitis	∙ Lesions are usually painful (rather than pruritic), last > 48 h, and leave discoloration on the skin
Systemic mastocytosis	∙ Rare condition that involves the internal organs (liver, spleen, lymph nodes, bone marrow), in addition to the skin
Atopic dermatitis	∙ Chronic, highly pruritic inflammatory skin disease ∙ Clinical manifestations vary with age
Bullous pemphigoid	∙ Chronic, autoimmune, blistering skin disease
Erythema multiforme	∙ Acute, self-limited, skin condition characterized by discrete targetoid lesions ∙ Considered to be a type IV hypersensitivity reaction to certain infections, medications, and other various triggers
Familial cold autoinflammatory syndrome	∙ Rare, inherited inflammatory disorder characterized by recurrent episodes of rash, fever/chills, joint pain, and other signs/symptoms of systemic inflammation triggered by exposure to cooling temperatures ∙ Onset usually occurs during infancy and early childhood and persists throughout the patient’s life
Fixed drug eruptions	∙ Lesions occur from exposure to a particular medication and occur at the same site upon re-exposure to the offending medication ∙ Lesions usually blister and leave residual pigmentation
Subacute cutaneous lupus erythematosus	∙ A non-scarring, photosensitive skin condition ∙ May occur in patients with systemic lupus erythematosus (SLE) and Sjögren syndrome
Pruritic urticarial papules and plaques of pregnancy	∙ Benign skin condition that usually arises late in the third trimester of a first pregnancy
Muckle-Wells syndrome	∙ Rare genetic disease that causes hearing loss and recurrent hives ∙ May lead to amyloidosis
Schnitzler's syndrome with monoclonal IgG kappa gammopathy	∙ Rare disease characterized by chronic, non-pruritic hives, periodic fever, bone and joint pain, swollen lymph glands and an enlarged spleen and liver

### Diagnostic tests

Skin prick tests (SPTs) and serum-specific IgE tests may help confirm a diagnosis of acute urticaria resulting from allergic or IgE-mediated (type I) reactions to common food allergens, latex hypersensitivity, stinging insect hypersensitivity and certain antibiotics. These tests are best performed by allergists with experience in interpreting test results in the appropriate clinical context. Random or screening tests for IgE to foods is discouraged given the high rate of false positive tests.

Certain diagnostic tests and assessments can be helpful in the diagnosis and differential diagnosis of CSU, including: a complete blood count (CBC), and erythrocyte sedimentation rate (ESR) or C-reactive protein (CRP) as markers of inflammation [6, 13, 14]. The presence of thyroid autoantibodies supports the autoimmune process in CSU. If there are atypical features, a skin biopsy, assessment of serum tryptase and complement levels, and serum protein electrophoresis should be considered. Current international urticaria guidelines recommend the following basic tests for all patients with CSU: differential blood count and CRP (to help rule out an underlying systemic disease) and total IgE and anti-thyroid peroxidase levels (which are important for predicting treatment response) [6].

Challenge testing, which reproduces exposure to a suspected stimulus in a supervised clinical environment, is often indicated to confirm a diagnosis of inducible urticaria. Cold-induced urticaria can usually be confirmed using the ice cube test (i.e., placing an ice cube in a sealed plastic bag over the forearm for 5–10 min, with urticaria developing upon rewarming of the skin). Dermatographism can be confirmed by lightly stroking the skin, or by using a standardized device such as a dermographometer. Aquagenic urticaria can be identified by immersion of a body part into lukewarm water or through the application of warm compresses. Hot bath testing and exercise challenge can help identify cholinergic urticaria, and the application of weight/pressure to the patient’s thigh or shoulder is helpful in the diagnosis of delayed-pressure urticaria [1, 2, 6].

## Treatment

**Fig. 3:**
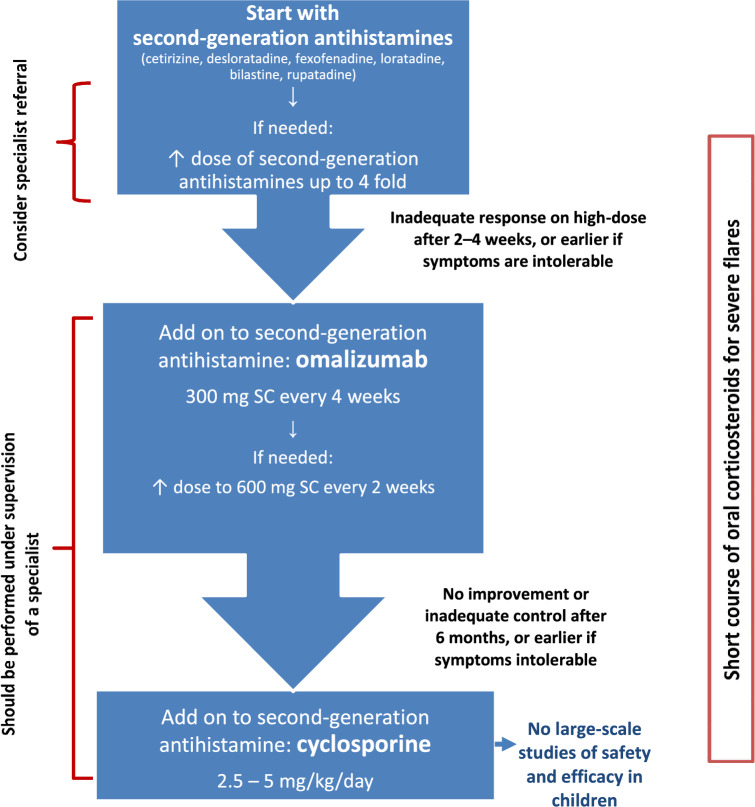
Simplified stepwise algorithm for the treatment of urticaria [6]. SC: subcutaneously. Adapted from Zuberbier 2022 [6]

Strategies for the management of urticaria include avoidance measures, antihistamines, and corticosteroids. Antihistamines are the mainstay of therapy. Corticosteroids should be used only in the short term for severe exacerbations given the risk of adverse events associated with prolonged use. For those patients with CSU who experience a poor response to antihistamines, omalizumab and cyclosporine are recommended as second-line and third-line treatments, respectively (see Fig. 3) [6].

### Avoidance

When a specific trigger can be identified in patients with acute urticaria (e.g., food, medication, insect venom, latex), strict avoidance is recommended. These patients should be provided with clear, written instructions on appropriate avoidance strategies and prescribed an epinephrine auto-injector (first-line management for anaphylaxis – please see *Anaphylaxis* article in this supplement) [2, 3]. These patients should be advised that exposure could not only lead to acute urticaria but also anaphylaxis.

For patients with CSU, NSAIDs, alcohol or opiates should be avoided as these can significantly exacerbate the condition. Food avoidance with elimination diets is not helpful for CSU.

### Medications

Second-generation, non-sedating H1-receptor antihistamines (i.e., fexofenadine, desloratadine, loratadine, cetirizine, bilastine, rupatadine; see Table 2) are the mainstay of therapy for urticaria. These agents have been shown to be significantly more effective than placebo for the treatment of urticaria [6, 13, 14]. First-generation antihistamines should be avoided due to their anticholinergic effects and associated side effects (i.e., sedation and cognitive impairment). Histamine type 2 (H2)-receptor blockers (such as ranitidine, famotidine and cimetidine) are not thought to be of benefit in the treatment of urticaria [28].

**Table 2:** Antihistamines commonly used and indicated for the treatment of urticaria

Second-Generation H1-receptor antihistamines	Standard adult dose	4 Times standard adult dose	Usual pediatric dose
Cetirizine (Reactine)	10–20 mg daily	40 mg daily	5–10 mL (1–2 teaspoons) daily (children’s formulation)
Desloratadine (Aerius)	5 mg daily	20 mg daily	2.5–5 mL (0.5–1.0 teaspoon) daily (children’s formulation)
Fexofenadine (Allegra)	120 mg daily	480 mg daily	Not currently indicated for children under 12 years of age
Loratadine (Claritin)	10 mg daily	40 mg daily	5–10 mL (1–2 teaspoons) daily (children’s formulation)
Bilastine (Blexten)	20 mg daily	80 mg daily	10 mg (1 oro-dispersible tablet or 4 mL oral solution) once daily for children ≥ 4 years weighing ≥ 16 kg
Rupatadine (Rupall)	10 mg daily	40 mg daily	5–10 mL (1–2 teaspoons) daily (children’s formulation)

Acute urticaria with unknown trigger(s) is usually treated with antihistamines at standard or higher doses. Guidelines for the management of CSU have recently been updated (see Fig. 3) [6]. A standard dose of a second-generation antihistamine is the initial treatment. Antihistamine efficacy is often patient specific and, therefore, more than one antihistamine should be tried before assuming therapeutic failure with these agents. Also, antihistamines are most effective if taken daily, rather than on an as-needed basis [29]. If symptoms are controlled with standard antihistamine doses, it is reasonable to continue treatment for several weeks to months, occasionally stopping therapy for brief periods to determine whether the urticaria has spontaneously resolved. In patients who do not achieve adequate symptom control at standard doses in 2–4 weeks, it is common practice to increase the antihistamine dose beyond the usual recommended dose [30]. Guidelines recommended up to four times the usual recommended dose of antihistamines in patients whose symptoms persist with standard therapy [6].

For patients with CSU who have not responded to four times the standard dose of second-generation antihistamines after a 2- to 4-week trial, omalizumab (an anti-IgE humanized monoclonal antibody), as add-on therapy is now considered the second-line option (see Fig. 3) [6]. Randomized double-blind, placebo-controlled trials have demonstrated its efficacy and safety in patients with CSU refractory to H1-antihistamines [31, 32]. Both 150 mg and 300 mg of omalizumab injected subcutaneously every 4 weeks have been shown to be effective [32].

Cyclosporine is considered third-line therapy if there is no response to omalizumab within 6 months, or if the condition is intolerable. Small, double-blind, randomized controlled trials have found cyclosporine (3–5 mg/kg/day) to be effective in patients with CSU who do not adequately respond to antihistamines [33, 34]. During treatment with cyclosporine, H1-receptor antihistamines should be continued, and blood pressure, renal function and serum cyclosporine levels should be monitored regularly given the significant side effects associated with this form of therapy (e.g., hypertension, renal toxicity).

For some patients with severe urticaria, a brief course of oral corticosteroids (e.g., 0.3–0.5 mg/kg of prednisone for 10–14 days) is warranted. However, long-term corticosteroid therapy should be avoided given the well-known side effects associated with prolonged use and the increased likelihood of developing tolerance to these agents.

The leukotriene receptor antagonist (LTRA), montelukast, can be used as an add-on to second-line treatment in H1-antihistamine refractory CSU. However, some clinical trials have not found the addition of this LTRA to be of significant benefit [35].

Various immunosuppressive or immunomodulatory therapies may provide some benefit for patients with severe CSU. Case reports and other small clinical trials have also found the following treatments to be effective for select patients with severe, refractory CSU: sulfasalazine, dapsone, hydroxychloroquine, colchicine, mycophenolate mofetil, 5-aminosalicylic acid, and intravenous immunoglobulin G (IVIG) [14]. However, the efficacy of these agents in the treatment of CU needs to be confirmed in large, randomized controlled trials. Also, it is important to note that these alternative treatments may have to be trialed given that anti-IgE therapy (omalizumab) is expensive and not available to all patients who fail high-dose, second-generation antihistamine therapy. More recently, remibrutinib—a novel, highly-selective, oral, covalent Bruton tyrosine kinase inhibitor—has been shown to be effective in CSU and to have a good safety profile [36].

QOL can be significantly impacted in patients with CSU. The condition can have a negative impact on work, school, social activities, diet and sleep. Instruments to monitor QOL (e.g., Chronic Urticaria-Quality of Life Questionnaire [CU-Q2oL]) and to quantify disease activity (e.g., the Urticaria Activity Score, Urticaria Control Test, Angioedema Control Test) have been developed and validated in adults and children [6]. These tools can also assist in monitoring response to therapies.

## Natural resolution

In adults, CU is reported to resolve spontaneously within 5 years in only 30–55% of patients [37]. In the pediatric population, resolution of CU is often defined as 1 year without symptoms in the absence of treatment [38]. Data on the natural history of CU and its subtypes in children are scarce. A Canadian study reported that the mean age at disease onset was 6.7 ± 4.7 years (range 0–17 years) [38]. Similar to adult studies, the resolution rate of CU in children was low, at 10.3 per 100 patients-years. The resolution rate for inducible forms is considered to be even lower; in one study, the resolution rate of cold urticaria in children was 4.8 per 100 patient-years [39].

## Specialist referral

While many cases of CU can be managed by primary-care physicians and pediatricians, there are certain situations when it is appropriate to refer the patient to an allergy specialist or dermatologist, including: failure of or difficulty tolerating first-line treatments; need for specialized treatment; and the presence of severe symptoms and/or atypical features (see Table 3). The decision to refer a patient with CU to a specialist should be based on shared decision-making between the patient and physician and should take into account the patient’s clinical presentation and treatment response. Consulting with an allergy or dermatology specialist can help ensure comprehensive management and optimal outcomes for patients with complex or refractory cases of urticaria.

**Table 3:** General considerations for specialist referral

A referral should be considered in any of the following situations: **1. Failure of or difficulty tolerating first-line treatments**. If the patient's CU persists despite the use of second-generation antihistamines at standard or four-times standard dosing, or if the patient is unable to tolerate high-dose second-generation antihistamines **2. Need for specialized treatments.** Specialists can recommend and have access to a wider range of treatment options for CU, including immunomodulatory therapies, in cases where standard treatments have been ineffective **3. Severe symptoms:** If the patient experiences severe symptoms of urticaria and angioedema, or if the condition significantly affects QOL **4. Atypical features:** If the presentation of CU is atypical or there are additional symptoms that raise concerns, such as systemic symptoms (e.g., fever, arthritis), a specialist can recommend a more comprehensive evaluation and appropriate diagnostic work-up	

## Conclusions

Urticaria is a common disorder that can be associated with angioedema. It is generally classified as acute (lesions occurring for < 6 weeks), chronic (lesions occurring for ≥ 6 weeks), spontaneous (no identifiable trigger) and inducible (lesions result from a specific trigger). The disorder can usually be diagnosed on the basis of clinical presentation and history; however, additional investigations may be helpful for confirming the diagnosis. Second-generation, non-sedating H1-receptor antihistamines represent the mainstay of therapy for both acute urticaria and CU, and up-dosing of these agents can result in better control for many individuals. First-generation antihistamines should be avoided due to their sedating and anti-cholinergic side effects. For severe CU, omalizumab and cyclosporine are considered second- and third-line therapies, respectively. Short courses of oral corticosteroids may be recommended for severe exacerbations, but long-term use is discouraged. Future studies investigating different targets related to mast cell activation may contribute to better control of severe/refractory cases.

## Data Availability

Not applicable.

## Abbreviations

CU: Chronic urticaria
CIndU: Chronic inducible urticaria
Ig: Immunoglobulin
QOL: Quality of life
CSU: Chronic spontaneous urticaria
SLE: Systemic lupus erythematosus
IVIG: Intravenous immunoglobulin G
NSAIDs: Non-steroidal anti-inflammatory drugs
ASA: Acetylsalicylic acid
SPTs: Skin prick tests
CBC: Complete blood count
ESR: Erythrocyte sedimentation rate
CRP: C-reactive protein
CU-Q2oL: Chronic Urticaria-Quality of Life Questionnaire
LTRA: Leukotriene receptor antagonist
H1: Histamine type 1
H2: Histamine type 2
